# Identification of Inflammatory Genes, Pathways, and Immune Cells in Necrotizing Enterocolitis of Preterm Infant by Bioinformatics Approaches

**DOI:** 10.1155/2021/5568724

**Published:** 2021-04-06

**Authors:** Lili Zhang, Lizhen Sun, Mingli Wu, Jie Huang

**Affiliations:** ^1^Neonatal Intensive Care Unit, Liaocheng Dongchangfu People's Hospital, Liaocheng 252000, China; ^2^Neonatal Intensive Care Unit, Liaocheng Dongchangfu Maternal and Child Health Care Hospital, Liaocheng 252000, China

## Abstract

**Background:**

Necrotizing enterocolitis (NEC) is one of the most serious gastrointestinal disease-causing high morbidity and mortality in premature infants. However, the underlying mechanism of the pathogenesis of NEC is still not fully understood.

**Methods:**

RNA sequencing of intestinal specimens from 9 NEC and 5 controls was employed to quantify the gene expression levels. RNA sequencing was employed to quantify the gene expression levels. DESeq2 tool was used to identify the differentially expressed genes. The biological function, pathways, transcription factors, and immune cells dysregulated in NEC were characterized by gene set enrichment analysis.

**Results:**

In the present study, we analyzed RNA sequencing data of NECs and controls and revealed that immune-related pathways were highly activated, while some cellular responses to external stimuli-related pathways were inactivated in NEC. Moreover, B cells, macrophages M1, and plasma cells were identified as the major cell types involved in NEC. Furthermore, we also found that inflammation-related transcription factor genes, such as STAT1, STAT2, and IRF2, were significantly activated in NEC, further suggesting that these TFs might play critical roles in NEC pathogenesis. In addition, NEC samples exhibited heterogeneity to some extent. Interestingly, two subgroups in the NEC samples were identified by hierarchical clustering analysis. Notably, B cells, T cells, Th1, and Tregs involved in adaptive immune were predicted to highly infiltrate into subgroup I, while subgroup II was significantly infiltrated by neutrophils. The heterogeneity of immune cells in NEC indicated that both innate and adaptive immunes might induce NEC-related inflammatory response.

**Conclusions:**

In summary, we systematically analyzed inflammation-related genes, signaling pathways, and immune cells to characterize the NEC pathogenesis and samples, which greatly improved our understanding of the roles of inflammatory responses in NEC.

## 1. Introduction

Necrotizing enterocolitis (NEC), one of the most serious gastrointestinal diseases in premature infants, is a significant cause of morbidity and mortality [1]. In North America, it affects about 1-3 per 1000 births annually [2]. Typical yet subtle signs of NEC in an infant include feeding intolerance, abdominal distention, and bloody stools, which could progress rapidly within hours [3]. Excessive inflammatory process in intestine, which could damage distant organs, is often observed in NEC patients, and NEC-affected infants are facing risks for developing microcephaly or suffering from neurodevelopmental delays [4]. However, the diagnosis of NEC largely depends on an abdominal X-ray and few breakthroughs are made in preventive strategies for NEC, regardless of considerable researches done in this field [5]. As nonspecific biomarkers in blood such as platelets and white blood cells could merely guide medical care for NEC patients, researchers have been exploring other markers to aid the early diagnosis of NEC and to measure the severity of it. Besides a spectrum of pro-/anti-/inflammatory mediators, such as serum amyloid A (SAA) and calprotectin, gut-associated specific biomarkers like trefoil factor-3 (TFF-3), claudin-3, and intestinal fatty acid-binding protein (I-FABP) have been widely introduced [6]. Notably, utilizing I-FABP in neonates studied between July 2005 and August 2010 could ensure a sensitivity of 93% and a specificity of 90% in assessing enterocyte damage [7]. Meanwhile, the investigation of the molecular mechanisms behind NEC has also promoted the understanding of this disease. Analyses of genome-wide expression profiles of bowel tissues from NEC patients have revealed several pathophysiological pathways that are related to inflammation, cell adhesion, extracellular matrix remodeling, and muscle contraction, triggered by a series of dysregulated genes [6], and the involvement of major transcriptional factors and growth factors are confirmed in NEC [8]. Moreover, several studies have indicated that gene polymorphism could also contribute to the development of NEC, but further investigation is still needed [9].

In the present study, we collected RNA sequencing data of human bowel tissues from nine NEC and five controls and conducted differential expression analysis, functional enrichment analysis, transcription factor activity prediction, and estimation of immune cell abundance, which revealed some key regulators, pathways, and immune cells involved in the pathogenesis of NEC.

## 2. Materials and Methods

### 2.1. The RNA Sequencing Data Analysis

The RNA-seq data with accession number SRP051825 was deposited in Sequence Read Archive (SRA, https://www.ncbi.nlm.nih.gov/sra) database [10]. The fastq files were downloaded from this database and, then, mapped to UCSC hg19 human genome by hisat [11]. The resulting bam files were used for quantifying the gene expression levels by Stringtie [12].

### 2.2. Differential Expression Analysis

The raw count data at gene level was used to identify the differentially expressed genes (DEGs), which was implemented in R package DESeq2 [13]. The thresholds of adjusted *P* value and fold change at 0.05 and 2 determined the DEGs [14].

### 2.3. The Dysregulated Pathways, Transcription Factors, and Immune Cells

The Reactome pathways were collected from MSigDB database [15], which curated a series of gene sets characterizing the pathways and target genes of transcription factors. Moreover, the immune cells were collected from previous studies [16, 17], which were used to quantify the tumor-infiltrating immune cells. The overrepresentation gene set enrichment analysis (ORA) was used to associate the dysregulated pathways, TFs, and immune cells with NEC. Which were implemented in R package clusterProfiler [18–20]. The threshold of adjusted *P* value was determined at 0.05.

### 2.4. The Hierarchical Clustering Analysis

The NEC cases and controls were clustered by the hierarchical clustering analysis based on the expression levels of dysregulated genes in NEC. Similarly, the two subgroups were also identified by the hierarchical clustering analysis and determined by the tree height with the largest difference.

### 2.5. Statistical Analysis

The statistical analysis was implemented in R programming tool. The two-sample comparisons were performed by Wilcoxon rank-sum test or student *t*-test. Multiple-sample comparisons were performed by analysis of variance (ANOVA) or Kruskal-Wallis test. *P* value of 0.05 was indicated as statistical significance.

## 3. Results

### 3.1. Samples from NEC and Controls

The RNA sequencing data of resected intestinal specimens of nine NEC and five controls who underwent bowel resection were downloaded from Sequence Read Archive (SRA) database, which was submitted by the researchers of the previous study [21], which took resected intestinal specimens of nine NEC and five controls who underwent bowel resection. The preterm patients who were diagnosed with stage III acute NEC and diseases other than NEC made up the case and control groups, respectively. The age (case vs. control: 27.42 vs. 32.02, *P* value = 0.07) and gender (female ratio case vs. control: 5/9 vs. 3/5, *P* value = 0.99) were not observed to have a difference between the two groups.

### 3.2. Identification of Dysregulated Genes in NEC

**Figure 1:**
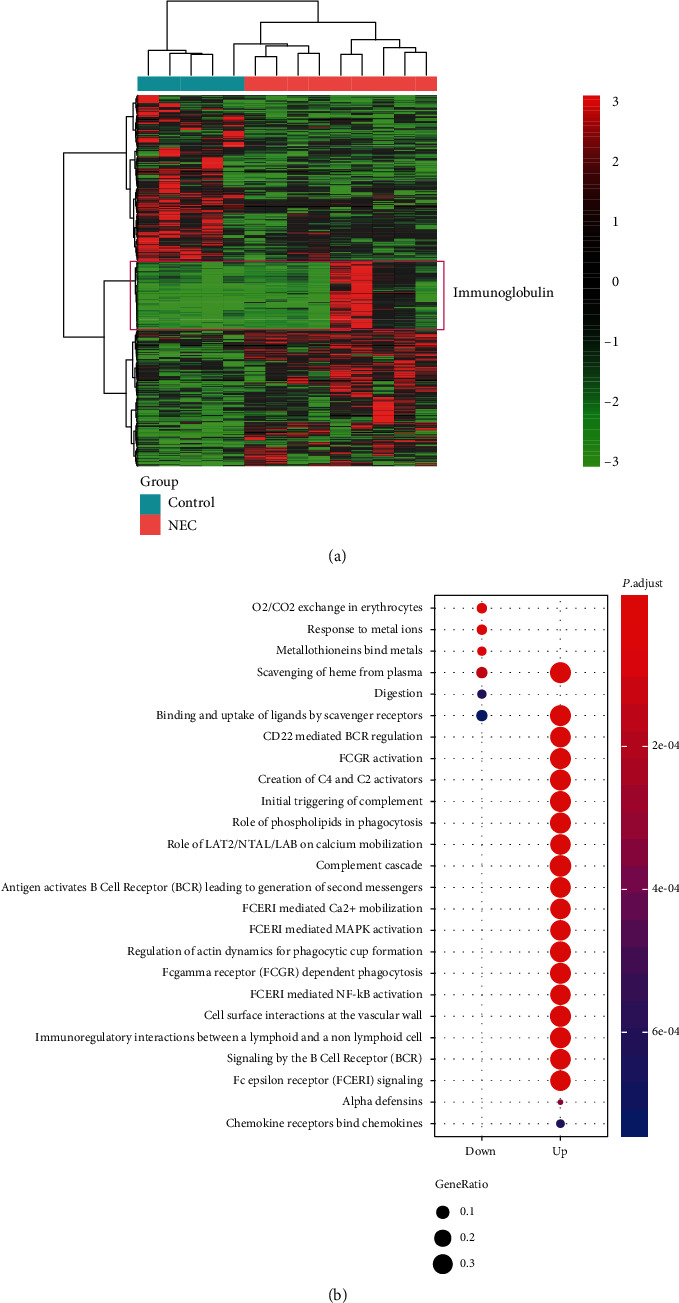
The expression patterns and biological function of differentially expressed genes (DEGs) in NEC. (a) The heat map displayed the expression patterns of the DEGs. A set of genes encoding immunoglobulin was highly upregulated in some of the NEC samples. (b) The Reactome pathways dysregulated in NEC. The pathways were enriched by the DEGs based on overrepresentation gene set enrichment analysis.

The differential expression analysis was conducted to identify the dysregulated genes in NEC. Totally, we identified 395 upregulated and 326 downregulated genes by comparing the gene expression profiles of NEC with those of controls (*P* value < 0.05 and fold change >2 or <1/2). As shown in Figure 1(a), the samples of NEC could be clearly differentiated from the controls by unsupervised clustering analysis. To characterize the biological function of these dysregulated genes, we conducted gene set enrichment analysis of the upregulated and downregulated genes, respectively. The Reactome pathways, such as signaling by the B Cell Receptor (BCR), immunoregulatory interactions between a lymphoid and a nonlymphoid cell, FCERI-mediated NF-*κ*B activation, antigen activates B Cell Receptor (BCR) leading to generation of second messengers, and CD22 mediated BCR regulation, were significantly enriched by the upregulated genes (Figure 1(b), adjusted *P* value < 0.05), suggesting that the dysregulation of immune system was a major hallmark of NEC. In accordance with these pathways, many genes encoding immunoglobulin were also found to be upregulated in NEC (Figure 1(a)). Moreover, we also observed some cellular responses to external stimuli-related pathways, such as metallothioneins bind metals, response to metal ions, and vesicle-mediated transport-related pathways, such as binding and uptake of ligands by scavenger receptors and scavenging of heme from plasma, were enriched by the downregulated genes (Figure 1(b)). In addition, the digestion was also observed to be downregulated in NEC, suggesting that the disease might cause the loss of digestion.

### 3.3. Identification of Immune Cells Involved in NEC

**Figure 2:**
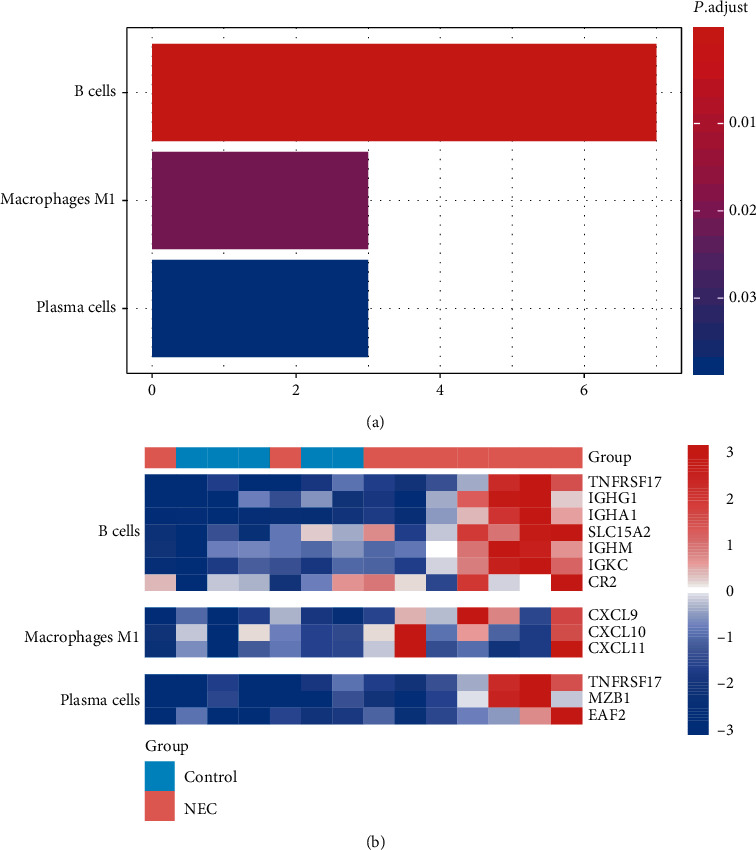
The immune cells infiltrating into the NEC samples. (a) The B cells, macrophage M1, and plasma cells were predicted to be activated in NEC samples. (b) The expression patterns of B cell, macrophage M1, and plasma cell-specific gene signatures in NEC and controls.

As described above, the upregulation of immune-related pathways and inflammation might be the hallmark of NEC. We then investigated the immune cells that participate in the pathogenesis of NEC. Two sets of marker genes for the immune cell were collected from previous studies [16, 17]. Based on the GSEA, B cells, macrophages M1, and plasma cells were identified as the major cell types involved in NEC (Figure 2(a)). urther analysis revealed that B cell-specific genes, such as TNFRSF17, IGHG1, IGHA1, SLC15A2, IGHM, IGKC, and CR2, macrophages M1-specific genes, such as CXCL9, CXCL10, and CXCL11, and plasma cells-specific genes, such as TNFRSF17, MZB1, and EAF2, were highly upregulated in NEC (Figure 2(b)). Notably, the macrophage M1-specific genes were chemokine ligands, suggesting that chemokine ligands were critical regulators in NEC pathogenesis.

### 3.4. Key Transcription Factors Involved in NEC

**Figure 3:**
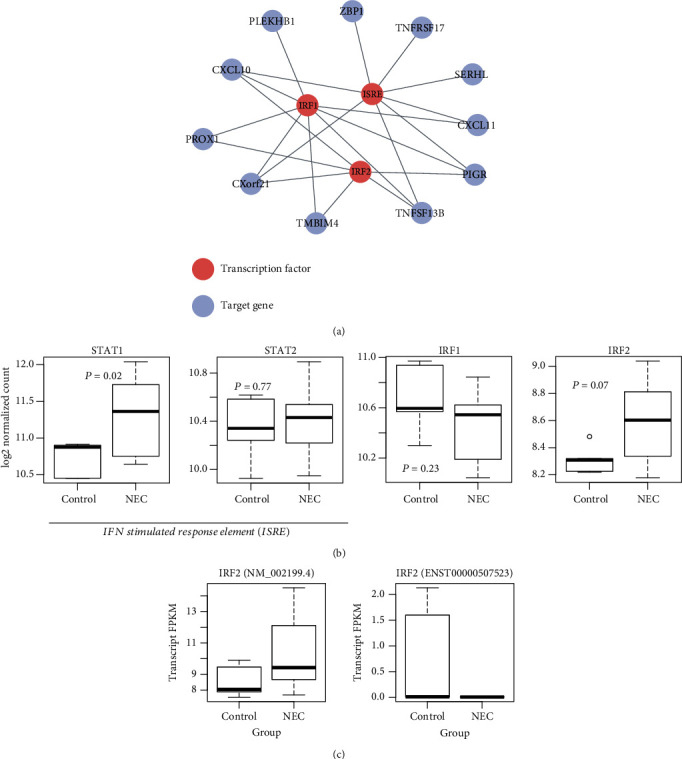
The dysregulated transcription factor (TF) regulatory network in NEC. (a) The TFs and target genes network in NEC. (b) The differential expression levels of TFs between NEC and controls. (c) Isoform switch event in two isoforms of IRF2.

To identify key transcription factors (TF) regulating the transcription of these dysregulated genes in NEC, we tested the degree of enrichment for the dysregulated genes in the TF targets. We found target genes of three TF or TF families, including *ISRE*, *IRF1*, and *IRF2*, were significantly enriched by the upregulated genes (Figure 3(a)). Notably, as IRF1 and IRF2 belonged to interferon regulatory factors, they jointly regulated the target genes encoding chemokine ligands, *CXCL10*, and *CXCL11* (Figure 3(a)). Moreover, most of these target genes, such as *CXCL10*, *CXorf21*, *TNFSF13B*, and *PIGR*, were regulated by all the three TFs.

Furthermore, we also investigated the expression patterns of the genes encoding the TFs. It should be noted that the ISRE TF family consisted of STAT1 and STAT2. We only observed STAT1 was significantly upregulated in NEC (Figure 3(b)), but the other genes encoding the TFs did not exhibit significant upregulation. However, IRF2 was slightly upregulated in NEC with a lower statistical significance (*P* = 0.07). To further investigate the expression patterns of IRF2 in NEC, we analyzed the expression levels of IRF2 isoforms. Remarkably, two of IRF2 isoforms were highly dysregulated in NEC, but only NM_002199.4, a major isoform of IRF2, was significantly upregulated in NEC (Figure 3(c)), suggesting that the protein encoded by NM_002199.4 was involved in the transcriptional regulation of the target genes.

### 3.5. Two Subgroups of NEC Identified by the Gene Expression Profiles

**Figure 4:**
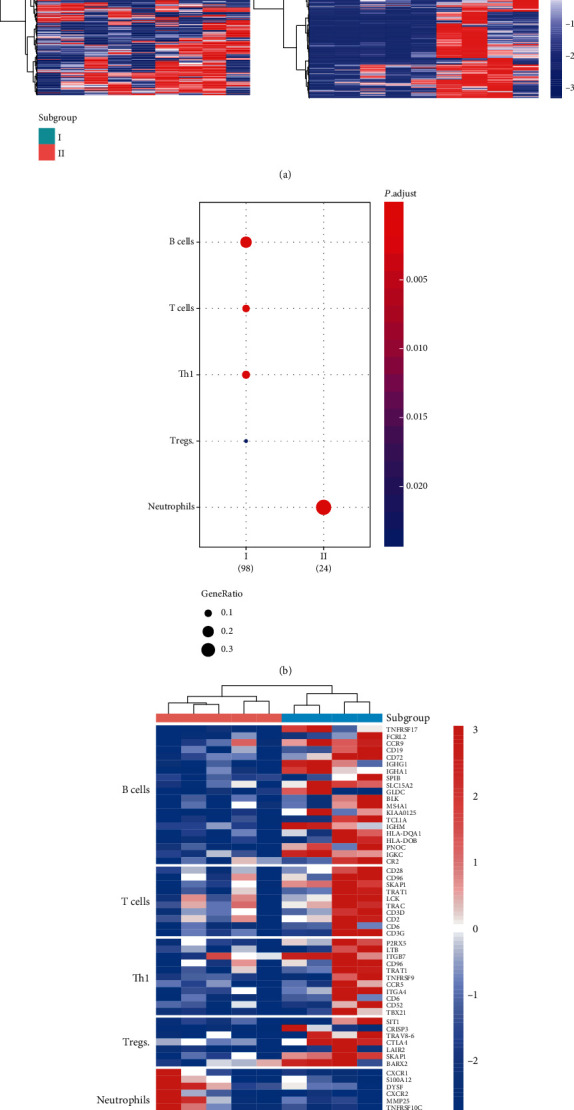
The two NEC subgroups based on gene expression profiles. (a) The expression patterns of DEGs in NEC (left) and between the two subgroups. (b) The immune cells infiltrating into two NEC subgroups. (c) The expression patterns of the genes specifically expressed in the immune cells.

As shown in Figure 1(a), the genes encoding immunoglobulin exhibited upregulated in only a subset of NEC patients, which gave us a hint that the NEC samples might exhibit heterogeneity to some extent. We then conducted unsupervised clustering analysis of the NEC samples based on the upregulated genes and identified two subgroups in NEC (Figure 4(a)). Similarly, the differential expression analysis was also conducted to identify the genes specifically dysregulated in either of the two subgroups (Figure 4(a)). As NEC was characterized by dysregulation of inflammatory response, we investigated the immune cells infiltrating into the two subgroups. Notably, B cells, T cells, Th1, and Tregs were predicted to highly infiltrate into the tissues of subgroup I, while subgroup II was significantly infiltrated by neutrophils (Figure 4(b), BH-adjusted *P* value < 0.05). Specifically, the signature genes of these immune cells were also observed to be dysregulated between the two subgroups. These results indicated that the heterogeneity in NEC-related inflammatory response is associated with both innate and adaptive immunes.

## 4. Discussion

Necrotizing enterocolitis is one of the most serious gastrointestinal diseases in premature infants, which causes high morbidity and mortality [1]. However, the underlying mechanism of the pathogenesis of NEC is still not fully understood. In the present study, we identified 395 upregulated and 326 downregulated genes in NEC samples, which could clearly distinguish the NEC from controls. Functional analysis of these dysregulated genes revealed that immune-related pathways were highly activated, while some cellular responses to external stimuli-related pathways were inactivated in NEC. The importance of immune response in NEC has been widely reported by previous studies [22, 23]. The attenuated cellular responses to external stimuli might result from the necrotic intestinal cells [24]. In addition, the digestion was also observed to be downregulated in NEC, suggesting that the disease might cause the loss of digestion.

Moreover, the comparison between NECs and controls identified immune cells and key transcription factors involved in NEC pathogenesis. Specifically, B cells, macrophages M1, and plasma cells were identified as the major cell types involved in NEC. Particularly, M1 macrophage had the potential to promote the progression of NEC [25], and necrotizing enterocolitis could be prevented by inhibiting M1 macrophage polarization [26]. Furthermore, we also found that inflammation-related TF genes, such as STAT1, STAT2, and IRF2, were significantly activated in NEC, further suggesting that these TFs might play critical roles in NEC pathogenesis.

Furthermore, we also found NEC samples might exhibit heterogeneity to some extent. Interestingly, two subgroups in the NEC samples were identified by hierachical clustering analysis (Figure 4(a)). Notably, B cells, T cells, Th1, and Tregs involved in adaptive immune were predicted to highly infiltrate into the tissues of subgroup I, while subgroup II was significantly infiltrated by neutrophils (Figure 4(b), BH-adjusted *P* value < 0.05). The innate immune has been widely studied in NEC; however, the underlying mechanism of immune cells involved in adaptive immune has not been well established [27–29]. The heterogeneity of immune cells in NEC indicated that both innate and adaptive immunes might induce NEC-related inflammatory response, and this difference in NEC samples might also be associated with the course of the disease.

Overall, the present study still has some limitations, such as small sample size, lack of experimental validation, and specific molecular mechanism. However, we systematically analyzed inflammation-related genes, signaling pathways, and immune cells to characterize the NEC pathogenesis and samples, which greatly improved our understanding of the roles of inflammatory responses in NEC.

## Data Availability

The data used in this manuscript can be found in SRP051825, which was deposited in Sequence Read Archive (SRA, https://www.ncbi.nlm.nih.gov/sra) database.
